# Caring-Related Chronic Low Back Pain and Associated Factors among Mothers of Children with Cerebral Palsy

**DOI:** 10.1155/2020/8854435

**Published:** 2020-12-30

**Authors:** Mehdi Ramezani, Jandark Eghlidi, Ehsan Pourghayoomi, Saeed Mohammadi

**Affiliations:** ^1^Department of Neuroscience, Faculty of Advanced Technologies in Medicine, Iran University of Medical Sciences, Tehran, Iran; ^2^Student Research Committee, Iran University of Medical Sciences, Tehran, Iran; ^3^Physiotherapy Research Center, Shahid Beheshti University of Medical Sciences, Tehran, Iran; ^4^Student Research Committee, School of Public Health, Iran University of Medical Sciences, Tehran, Iran

## Abstract

**Background:**

Literature indicated some risk factors for low back pain; however, there is insufficient knowledge on the effect of caring-related physical activities and individual characteristics on Chronic Low Back Pain (CLBP) in mothers of children with Cerebral Palsy (CP).

**Objective:**

The main aim of the current study was to determine the association between caring-related physical activities, Body Mass Index (BMI), education level, and CLBP in mothers of children with CP.

**Design:**

Case-control observational study. *Setting*. Pediatric rehabilitation clinics. *Participants*. Mothers of children with CP. *Main Outcome Measures*. Measures is comprised of a self-administered questionnaire that included the demographic characteristics items, pain visual analog scale, and three items of the job-related physical demands questionnaire. The logistic regression model served to assess the association.

**Results:**

The control group included 81 healthy mothers, with a mean (SD) age of 39 (8.45) years, and the case group contained 90 mothers who suffered from CLBP, with a mean (SD) age of 37 (8.64) years. Performing lifting movements (OR 13.73, *β* = 2.62, *p* < .001), BMI (OR 11.85, *β* = 2.47, *p* = .011), repetitive bending (OR 7.67, *β* = 2.04, *p* = .010), forward-flexion (OR 6.71, *β* = 1.91, *p* = .033), and level of education (OR .21, *β* = −1.53, *p* = .020), in descending order of odds ratios, were found to be significant predictors of the CLBP in mothers of children with CP.

**Conclusion:**

Avoiding caring-related harmful physical activities, maintaining body weight within a healthy range, and increasing knowledge for accurate lifting/handling techniques can be helpful to prevent the CLBP in mothers of children with CP.

## 1. Introduction

Chronic Low Back Pain (CLBP) is defined as pain localized below the costal margin and above the inferior gluteal folds, with or without leg pain, for longer than three months [1, 2]. CLBP is the most prevalent caring-related disability in mothers of children with Cerebral Palsy (CP) (44.7%) [3, 4]. Also, it is the most common cause of limitations in these mothers and causes undesirable effects on the quality of life [5–8].

Some risk factors for low back pain have been reported in different occupational groups. In previous studies, it has been demonstrated that the high grade Body Mass Index (BMI) [9, 10] and the low level of education [8, 9, 11, 12], as individual characteristics, lead to low back pain. Also, it has been reported that bending [4, 9, 13], lifting [9, 10, 13–15], and repetitive movements [4, 9, 16] lead to low back pain as work-related physical factors. In this regard, caring-related activities require different movements such as bending, twisting, lifting, and frequent flexing/extending of body parts, mainly around the back and lumber. These movements induce physical stresses that may lead to injuries in many cases [17–21]. Based on our best knowledge, no evidence indicated the precise impact of caring-related activities, BMI, and level of education on CLBP in mothers of children with CP.

The main aim of the current study was to determine the association of caring-related physical activities, BMI, and education level on CLBP in mothers of children with CP. Precisely identifying the risk factors of CLBP can be helpful for timely prevention and improve the quality of life and caregiving in mothers of children with CP [4, 22].

## 2. Materials and Methods

### 2.1. Participants

Data of the present observational case-control study was collected at university-based pediatric rehabilitation clinics, Tehran, Iran, from August 2015 to January 2019. Mothers of children with CP have applied for and received rehabilitation services for their children. The following formula (Equation (1)) [23] at a confidence level of 95% (*α* = .05), incidence (*ρ*) of .45, and absolute error (*d*) of .05 was used to calculate the sample size, with the convenient sampling method. The optimum sample size was calculated at 379. However, only about 300 cases were received rehabilitation services in clinics during the study. Accordingly, after using finite population correction, 379 ÷ (1 + (379 ÷ 300)), the required sample size was obtained at least 168.

(1) $$\begin{array}{c} N=Z{\left(\left(1-\alpha \right)÷2\right)}^{2}\times \rho \left(1-\rho \right)÷d^{2}. \end{array}$$

The recruited participants (*N* = 171) were assigned into two groups: the control group (mothers without CLBP; *n* = 81) and the case group (mothers with CLBP; *n* = 90). Two groups were homogenized for the age, type of work (all mothers were a housewife and no one had an official job), and transferability of the child (all CP-children needed to assist with the transfer [4]), as potential confounders. All participants were primary caregivers of their CP-children, 2 to 10 years old. The inclusion criteria were the history of pain in the lumbar and/or buttock region (defined as pain reported below the level of T12 and no lower than the buttock line) for longer than three months in participants of the case group, no pain from the past 12 months for participants in the control group, older than 18 years old, and having at least 7 months of experience in taking care of the child [3, 4, 9, 24–26]. The exclusion criteria were signs of serious spinal pathology (red flags) including significant trauma, unexplained weight loss and widespread neurologic changes, current radiating symptoms (and/or neurological deficit) below the level of the buttock crease, current pregnancy or breastfeeding, history of spinal surgery, fracture or malignancy, and mothers caring for more than one child with disabilities [3, 24, 25, 27]. All participants were provided informed consent before participating in the research. The current study followed the principles of the Declaration of Helsinki. Also, the protocol of the study was approved by the Research Board and the Committee of Medical Ethics, Shahid Beheshti University of Medical Sciences, Tehran, Iran (IR.SBMU.RETECH.1394.90).

### 2.2. Study Measures

Data were collected using a self-administrated questionnaire that included demographic items (age and education level), Visual Analog Scale (VAS), and selected three items of the Job-Related Physical Demands (JRPD) questionnaire. The recall bias may have occurred in the case-control approach [28]. However, based on administrative purposes for collecting data, by using highly valid scales, we reduced the recall bias [28, 29].

The VAS is commonly used in pain studies and is an optimal tool for describing pain severity or intensity [30]. It is a 100 mm horizontal line in which the patient remarks her pain intensity. The range of score is from zero (no pain at all) to 100 (worst pain imaginable) [31]. For participants in the case group, the scores of ≤5 mm were considered no pain [30, 32]. Also, the formula of the *weight in kilograms divided by the square of the height in meters* was used to calculate the BMI. It usually classifies into three grades: the high grade (overweight) covers scores equal to or more than 25, the normal grade covers scores between 20.1 and 25, and the low grade (underweight) covers scores equal to or less than 20 [33]. For calculating BMI, a weighing scale and metal tape were used to measure body weight and height, respectively. Finally, the education level was categorized into two levels: the academic/high (above the diploma) level and the nonacademic/low (diploma and below) level [9, 34].

There are limited tools for easily assessing caring-related physical requirements [10, 35]. However, the JRPD is a highly valid self-report questionnaire to measure the frequency of movements, such as bending, twisting, lifting, sitting, and activities that are consistently found to be related to low back pain [9, 10, 36]. The JRPD questionnaire has 38 items that examine both types of exposure and the duration of such exposures [26]. Internal consistency and reliability were reported as excellent in both the original and Persian versions of JRPD [9, 26, 36]. In the current research, three items of the JRPD with good test-retest reliability [9] were selected based on the reported physical risk factors for low back pain in other occupational groups [17–19]. The selected items included the following: (1) *forward-flexion* movements—I lean forward continually when I work (when sitting, when standing, when pushing carts, etc.); (2) *repetitive bending*—I repeatedly bend my back (e.g., forward, backward, to the side, or twist) in the course of my work; and (3) *lifting movements*—I lift materials that weight more than 25 pounds. Participants were asked to answer each item in five-point Likert-type scales: 1 (never), 1 (≤5 hours/week), 2 (≤2 hours/day), 3 (2 to 4 hours/day), and 4 (≥4 hours/day) [26]. In the present study, based on the scoring method in JRPD (assigning the same score of 1 for scales of *never* and *≤5 hours/week*), we categorized the answers in two levels: the level of *Not-Doing* activity (scales of never and ≤5 hours/week) and *Doing* activity (scales of ≤2 hours/day, 2 to 4 hours/day, and ≥4 hours/day).

### 2.3. Statistical Analysis

The mean and standard deviation (SD) of the quantitative variables and the frequency (%) of the qualitative variables were reported. The normality was checked using the Shapiro–Wilk test. The group differences for numerical and categorical variables were analyzed by the independent samples *t*-test and chi-square test, respectively. A multiple logistic regression model served to assess the association. The statistical significance was defined as *p* < .05, and the confidence interval was considered at 95%. SPSS, version 25 (IBM Corporation) was used to analyze data.

## 3. Results

The control group contained 81 healthy mothers, with a mean (SD) age of 39 (8.45) and a range of 22 to 65 years. The case group included 90 patients, with a mean (SD) age of 37 (8.64) and a range of 22 to 60 years. Two groups were homogenized for the age (*p* > .05). Participants in the case group had pain intensity of more than 5 VAS scores. However, participants in the control group, as healthy subjects, had no pain during the study (VAS scores = 0). The frequency of the nonacademic education level was 25.9% in the control group and 63.3% in the case group. The frequency of high-grade BMI was 9.9% in the control group and 64.4% in the case group (Table 1).

The study groups had differences in BMI, education level, and all three selected physical activities (Table 1). Hence, the final model of multiple logistic regression was analyzed using these five variables. Results of the logistic regression analysis indicated that lifting movements (OR 13.73; *p* < .001), being overweight (OR 11.85; *p* = .011), repetitive bending (OR 7.67; *p* = .010), lumbar forward-flexion movements (OR 6.71; *p* = .033), and low level of education (OR 0.21; *p* = .020), in descending order of odds ratio, were associated with CLBP in mothers of children with CP (Table 2).

## 4. Discussion

As anticipated, the results of logistic regression analysis showed that the selected physical activities along with the overweight and low level of education are contributors to suffering from CLBP among the mothers of CP-children.

The results of the present study indicated that mothers who performed lifting movements had a 13.73 times higher chance of developing CLBP than those who did not. Previous studies have been confirmed that lifting movements are associated with low back pain [10, 14]. Coenen and colleagues [15] investigated the effect of lifting during work on the incidence of low back pain in occupational populations exposed to lifting. They concluded that the intensity and frequency of lifting could significantly increase the annual incidence of low back pain [15]. In lifting, excessive forces on the spine and overactivation of the paraspinal muscles lead to muscle fatigue. Following muscle fatigue, forces transfer to the passive structures such as discs between the vertebrae and lead to degeneration of discs [37]. Therefore, safe handling and lifting techniques can probably prevent CLBP.

In the current study, in comparison to underweight mothers, overweight mothers had an 11.85 times higher chance of developing CLBP. Being overweight/obese is a factor strongly associated with disc degeneration and increases the severity of disc degeneration [38]. Overweight/obese subjects generally have a high blood level of cholesterol and fat, which can cause atherosclerosis and reduce the blood supply to the intervertebral discs [39]. Therefore, overweight can increase the chance of discs injuring. Also, overweight persons are at risk of osteoarthritis in weight-bearing joints [40]. The excessive physical loads on vertebrae can cause conditions such as excessive wear and tear between vertebras and lead to osteoarthritis [40]. Although the majority of studies confirmed the association between overweight and low back pain [9, 10, 38, 40–44], there is insufficient evidence to establish a direct causal link between overweight and low back pain.

Our findings indicated that mothers who performed repetitive bending had a 7.67 times higher chance of developing CLBP than those who did not. In line with our result, the association of repetitive bending with low back pain has been confirmed in previous studies [10, 45]. Moreover, by selecting the forward-flexion movements, we considered the association of care-related forward flexing of the lumbar with CLBP, e.g., during various positions and while pushing a baby wheelchair, stroller, or other similar movements. The odds ratio for this item was obtained at 6.71. Accordingly, mothers who performed these types of activities had a high chance of developing CLBP. Also, the results of previous studies in various occupational groups are in agreement with our findings [10, 14]. There is a specific lumbopelvic movement pattern that may play an essential role in maintaining the chronicity in low back pain [42]. It seems that the pain-free individuals, but not those with CLBP, could sufficiently regulate the lumbopelvic movement pattern while bending tasks executed at various speed levels. Individuals with CLBP probably showed a stereotyped strategy at their lumbar spine and hip joints [42]. Also, Esola and colleagues found significant differences in the pattern of motion between their case and control study groups' [43]. Patients in their case group had a significantly lower lumbar-to-hip flexion ratio during the middle forward bending [43]. Therefore, performing forward-flexion activities may help to maintain the chronicity in patients with CLBP because of the insufficient lumbopelvic and/or lumbar-to-hip movement pattern [42, 43].

In the present study, the low level of education was found to be a risk factor for CLBP. Fifty-seven out of 78 mothers with nonacademic education level were suffered from CLBP. The real mechanisms of linking between the level of education and back pain are still obscure [46]. Nonetheless, Dionne and colleagues confirmed that the low education status is significantly associated with an increased prevalence of back pain and a higher point prevalence of signs and symptoms [11]. Batista and colleagues were also concluded that individuals with a high education level were less affected by low back pain than those with medium or low educational levels [12]. The previous studies have been demonstrated that subjects with high education exhibit better performance in managing daily living activities, keeping a balance among movement and rest, avoiding potentially harmful activities and repetitive movements, and having a sufficient calcium intake [46, 47]. Therefore, it can be concluded that the high level of education is a protective factor for the CLBP in the mothers of children with CP. Also, it has been indicated that back-related educational programs reduce the prevalence of low back pain among different populations such as nurses [48–50]. Therefore, increasing knowledge about the accurate lifting/handling techniques and maintaining body weight within a healthy range may prevent suffering from CLBP among mothers of children with CP.

Based on our best knowledge, this is the first study to investigate the caring-related factors associated with CLBP in mothers of children with CP. Therefore, there is no sufficient evidence to support our findings in the mothers of CP-children. However, we have compared the results of the present study with those of other occupational groups. In line with our findings, performing harmful physical activities [9, 34, 51, 52], such as frequent bending [9, 52] and heavy lifting [9, 51], are reported as risk factors to progress the chronicity of low back pain in nurses, industrial workers, and military forces. High BMI has been also reported as a risk factor for CLBP in industrial workers and military forces [9, 34]. In contrast to our findings, the education level was not reported as a risk factor for CLBP in industrial workers [34]. However, a population-based national study in Iran has confirmed the role of low education in the progress of CLBP [53]. This study [53] has also confirmed the role of high BMI and high-load physical activities in the CLBP of the Iranian population.

This study has some limitations, such as using a convenient sampling method and self-reported measures. Also, in the present study, we have entered the caring-related physical demands and some individual characteristics in the logistic regression model. In future studies, therefore, we suggest considering the effect of psychological aspects besides the physical and individual characteristics.

## 5. Conclusions

In conclusion, the results of the current study confirmed the association of performing harmful physical activities (lifting, repetitive bending, and lumbar forward-flexion), overweight, and low level of education with CLBP. Avoiding harmful physical activities, maintaining body weight within a healthy range, and increasing knowledge about the accurate lifting/handling techniques can be helpful for the prevention of CLBP in mothers of children with CP.

## Figures and Tables

**Table 1:** Comparing mean (standard deviation) and frequency (*%*) of the demographic and clinical assessments.

Variable		Case *n* = 90	Control *n* = 81	Total *N* = 171	Group differences	
Age (y)		37 (8.64)	39 (8.45)	38 (8.62)	*t* = −1.92	.060
Visual analog scale (mm)		61 (21.89)	0^∗^	32 (34.61)	*t* = 25.63	**<.001**
Education level	Academic	33 (36.70)	60 (74.10)	93 (54.40)	*X* ^2^ = 24.04	**<.001**
	Nonacademic	57 (63.30)	21 (25.90)	78 (45.60)		
Body Mass Index	High	58 (64.40)	8 (9.90)	66 (38.60)	*X* ^2^ = 58.59	**<.001**
	Normal	23 (25.60)	66 (81.50)	89 (52.00)		
	Low	9 (10.00)	7 (8.60)	16 (9.40)		
Forward flexion	Doing	64 (71.10)	18 (22.20)	82 (48.00)	*X* ^2^ = 40.82	**<.001**
	Not-Doing	26 (28.90)	63 (77.80)	89 (52.00)		
Repetitive bending	Doing	68 (75.60)	8 (9.90)	76 (44.40)	*X* ^2^ = 74.48	**<.001**
	Not-Doing	22 (24.40)	73 (90.10)	95 (55.60)		
Lifting movements	Doing	61 (67.80)	11 (13.60)	72 (42.10)	*X* ^2^ = 51.37	**<.001**
	Not-Doing	29 (32.20)	70 (86.40)	99 (57.90)		

^∗^No one in the control group had pain during the study. Bolded items represent statistical significance.

**Table 2:** Comparison of multiple logistic regression outcomes.

Variable		*β*	SE	*p* value	OR	95% CI for OR	
						Lower bound	Upper bound
Education level		-1.53	.64	**.020**	.21	.61	.76
^∗^Body Mass Index	High	2.47	.97	**.011**	11.85	1.77	79.33
	Normal	-1.68	1.02	.101	.18	.02	1.39
Forward flexion		1.91	.89	**.033**	6.71	1.17	38.58
Repetitive bending		2.04	.78	**.010**	7.67	1.66	35.43
Lifting movements		2.62	.73	**<.001**	13.73	3.25	57.93

*β*: beta; OR: odds ratio; SE: standard error; CI: confidence interval. ^∗^Lower grade of body mass index as reference category. Bolded items represent statistical significance.

## Data Availability

Data of the present study are restricted by the Physiotherapy Research Center, Shahid Beheshti University of Medical Sciences, in order to protect patient privacy. Data are available from Mehdi Ramezani (mramezaniiiiii@gmail.com) for researchers who meet the criteria for access to confidential data.
